# A new species of *Elpidium* (Crustacea, Ostracoda) from bromeliads in Cusuco National Park, Honduras

**DOI:** 10.3897/zookeys.313.4904

**Published:** 2013-07-01

**Authors:** Ricardo L. Pinto, Merlijn Jocqué

**Affiliations:** 1 University of Brasília, Institute of Geosciences, Laboratory of Micropaleontology, ICC-Ala Central, Subsolo ASS339/10, Campus Universitário Darcy Ribeiro, Asa Norte, Brasília/DF, CEP 70910-900, Brazil; 2 Koninklijk Belgisch Instituut voor Natuurwetenschappen (KBIN), Vautierstraat 29, 1000 Brussel, Belgium; 3 Operation Wallacea, Hope House, Old Bolingbroke, Lincolnshire, UK; 4 Institute for Biodiversity and Ecosystem Research, Bulgarian Academy of Sciences, 2 Yuri Gagarin Street, 1113 Sofia, Bulgaria

**Keywords:** Timiriaseviinae, phytotelmata, dispersal, endemism, neotropics, cloud forest

## Abstract

Passively dispersing aquatic invertebrates such as Ostracoda in restricted aquatic habitats such as bromeliads remain an intriguing observation considering the highly specialised dispersal vectors needed for efficient colonisation. Here we describe a new species of *Elpidium*, *Elpidium merendonense* **sp. n.**, collected from bromeliads in the cloud forest from Cusuco National Park, Honduras. *Elpidium merendonense* **sp. n.** is a small to medium-sized species that can be easily distinguished from its congeners by its unique outgrowth at the posterior end of the left valve, visible especially in females. The species was common all through the park occurring at a wide range of altitudes and in different species of bromeliads. This finding is the first freshwater ostracod species described from Honduras and is in agreement with the prediction that the genus *Elpidium* contains a large number of species with small geographic distributions. We update the list of described species of *Elpidium* and present a key to species.

## Introduction

Phytotelmata are plant structures that hold water, such as tree holes, flowers, husks or leaf brackets. These water bodies are completely rain dependent and often contain communities of highly specialised aquatic invertebrates (Kitching 2000). Well known examples of phytotelmata are several species of Bromeliaceaea, a group of terrestrial and epiphytic perennial plants distributed from Argentina to Florida, and with one representative in Africa. The bromeliads that contain enough water to house aquatic invertebrate communities are called tank bromeliads. As a consequence of their architecture, bromeliads provide high quality living space in the form of humus and moisture for diverse biota (Benzing 2000).

The first discovery of an ostracod species in bromeliad tanks was published by Fritz Müller (1880), based on material collected in the state of Santa Catarina, Southern Brazil. A new genus and species were then described, *Elpidium bromeliarum* Müller, 1880, and one year later an extension of the species description was published (Müller 1881). A putative record of this species was later reported from Costa Rica (Picado 1913). Since then, other species in the genus have been described from the Caribbean, all of them from bromeliad phytotelmata. Tressler (1941) described *Elpidium maricaoensis* (Tressler, 1941) from Puerto Rico. After studying material from Jamaica, Tressler (1956) described *Elpidium laesslei* (Tressler, 1956) and recorded *Elpidium bromeliarum* from that country. Pinto and Purper (1970) published a revision of the genus, wherein a re-description of the type species was provided with the designation of a neotype, since the original type series had been lost. Three new species were described from Cuba (Danielopol 1975) but were left in open nomenclature (*Elpidium* sp. A, *Elpidium* sp. B and *Elpidium* sp. C). These three Cuban species were later named *Elpidium inaequivalve* Danielopol, 1980, *Elpidium pintoi* Danielopol, 1980 and *Elpidium purperae* Danielopol, 1980 respectively (Colin and Danielopol 1980).

Studies on *Elpidium* remained predominantly taxonomic until relatively recently, when Little and Hebert (1996) investigated the evolutionary ecology of *Elpidium* ostracods in Jamaica: nine species could be distinguished based on morphological and genetic evidence, but none of these were formally described. Additionally, most species showed restricted distribution (high endemism) and only rarely species co-occurred, which led the authors to conclude that bromeliads seem to be a source of high biodiversity, working as ‘ecological islands’ that lead to frequent allopatric speciation events (Little and Hebert 1996). These results suggest that the diversity of the genus, with its current six valid species, is strongly underestimated and that the whole area of distribution of the genus has been thus far poorly investigated.

In the present work, we report on a new species of *Elpidium* from Cusuco National Park, which consists of the first ostracod species collected from bromeliad phytotelmata in Honduras.

## Material and methods

Material used for the species description was collected during the survey of aquatic invertebrate communities in bromeliads from 12th July to 22nd August 2006 in Cusuco National Park (CNP), Honduras. This survey is part of the yearly biodiversity survey by the ecovolunteer driven conservation organisation Operation Wallacea (UK) in CNP. CNP is situated in north-western Honduras, within the Merendón Mountain range. The core zone of the park consists of lower montane tropical rain forest (a mix of primary and secondary), with patches of primary cloud forest and upper montane rain forest characterised by high densities of bromeliads. Bromeliads sampled for Ostracoda were *Tillandsia guatemalensis* Smith and *Catopsis* sp. We sampled a total of 110 bromeliads in 12 different sites in the park, for a more detailed description of the study of aquatic invertebrates in bromeliads we refer to the protocol described in Jocque et al. (2010). The material in this study comes from a single bromeliad (*Tillandsia guatemalensis*) in the core zone of the park. The bromeliad was attached at about 1.5 meter above the ground. The collected plants were dismantled leaf by leaf and ostracods were collected from the water in the bromeliads and preserved in 70% ethanol.

For description, specimens were dissected under a stereomicroscope; valves were stored dry in micropaleontological slides and soft parts were mounted in permanent slides with CMC-9AF mounting medium (Masters Company Inc., Bensenville, Illinois, USA). Micrographs of valves and carapaces were obtained with a scanning electron microscope; line drawings of appendages and body parts were made under an optical microscope with the aid of a camera lucida.

All analysed material is deposited in the crustacean collection of the Museu de Zoologia da Universidade de São Paulo (MZUSP).

Higher taxonomy of the Ostracoda follows the synopsis by Horne et al. (2002).

## Taxonomy

### Class Ostracoda Latreille, 1802 Subclass Podocopa G.W. Müller, 1894 Order Podocopida G.O. Sars, 1866 Suborder Cytherocopina, Baird, 1850 Superfamily Cytheroidea Baird, 1850 Family Limnocytheridae Klie, 1938 Subfamily Timiriaseviinae Mandelstam, 1960

#### Genus Elpidium

F. Müller, 1880

http://species-id.net/wiki/Elpidium

##### Type species

(by original designation): *Elpidium bromeliarum* F. Müller, 1880.

**Other species allocated:** *Elpidium inaequivalve* Danielopol, 1980; *Elpidium laesslei* (Tressler, 1956) Danielopol, 1980; *Elpidium maricaoensis* (Tressler, 1941) Danielopol, 1980; *Elpidium pintoi* Danielopol, 1980; *Elpidium purperae* Danielopol, 1980.

##### Diagnosis.

Medium sized to relatively large ostracods; carapace broad, generally bigger in width than in height; ventral margin flat; pale to dark brown smooth surface; in dorsal and ventral view males with greatest width at midlength, females with posterior part expanded into a brood pouch carrying eggs and greatest width displaced posteriorly; strongly interlocking selvages along ventral margin leaving an anteroventral gap between left and right valve margins; hinge a crenulated cardinal bar on the smaller valve forming rudimentary anterior and well developed posterior teeth; A1 with 5 functional articles, the first one bearing a sub-apical expansion with a tuft of tiny setules on the dorsal margin; in males, A2 with a serrated apical claw on the terminal segment (no such serration in females); terminal segment of A2 with a small lobe (hyaline formation) in both males and females; second and third endites of the maxillule bearing two spatulate claws each; copulatory process of hemipenis a hook-like structure placed ventrally on the muscular body, near the base of distal lobe.

##### Comparison between *Elpidium* and *Intrepidocythere*.

*Elpidium* is closely related (Pinto et al. 2008) to the terrestrial genus *Intrepidocythere* Pinto et al., 2008. Nonetheless several differences can be recognized. The carapace of *Intrepidocythere* is smaller and considerably less broad in dorsal view compared to *Elpidium* species. Furthermore, the marginal zone and hinge structures are different, while *Elpidium* has a long bar on the smaller valve ending in a small anterior tooth and a crenulated posterior tooth, *Intrepidocythere* has a smooth medial ridge and a posterior socket in left valve with the complementary smooth medial groove and posterior tooth in right valve. The antennule has 2 medio-dorsal setae on the fourth segment in *Elpidium*, but only 1 in *Intrepidocythere*; the male antenna has 1 serrated claw on the terminal segment in *Elpidium*, but 2 in *Intrepidocythere*; and the female caudal ramus has three setae in *Elpidium*, but only two in *Intrepidocythere*.

#### Elpidium merendonense sp. n.

urn:lsid:zoobank.org:act:0300306E-1A75-4EB3-8C43-2221036F40A1

http://species-id.net/wiki/Elpidium_merendonense

**Figure 1.**
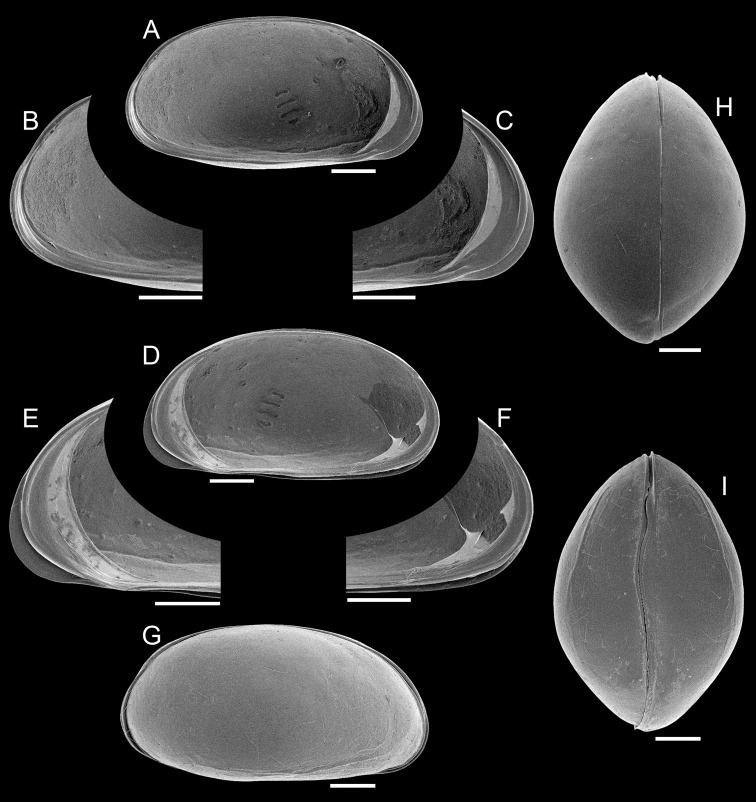
*Elpidium merendonense* sp. n., male. **A** Left valve, internal view **B** left valve, internal view, detail of postero-ventral margin **C** left valve, internal view, detail of antero-ventral margin **D** right valve, internal view **E** right valve, internal view, detail of antero-ventral margin **F** right valve, internal view, detail of postero-ventral margin **G** right lateral view **H** dorsal view **I** ventral view. **A–F** holotype, MZUSP 29072; **G** paratype, MZUSP 29077; **H** paratype, MZUSP 29078; **I** paratype, MZUSP 29079. Scale bars: 100 µm.

Figs 1 [Fig F2] [Fig F3] –4

##### Type locality.

Cusuco National Park, Honduras at an altitude of 1840 masl. Geographical coordinates: 15.5133N, 88.2417W. Water accumulated in bromeliad (*Tillandsia guatemalensis*) leaf axils. Material collected on 15 July 2006 by M. Jocqué.

##### Type material.

Holotype: a dissected male, with valves dried and coated for scanning electron microscopy stored in a micropaleontological slide and soft parts mounted in a permanent slide with CMC-9AF mounting medium (MZUSP 29072). Allotype: a dissected ovigerous female, with valves stored dry in a micropaleontological slide and soft parts mounted in a permanent slide with CMC-9AF mounting medium (MZUSP 29073). Paratypes: two males (MZUSP 29074 and MZUSP 29075), dissected and stored like the allotype; an ovigerous female (MZUSP 29076) dissected and stored like the holotype; three males (MZUSP 29077, MZUSP 29078 and MZUSP 29079) and three females (MZUSP 29080, MZUSP 29081 (carapace broken) and MZUSP 29082), dried and coated for scanning electron microscopy stored in micropaleontological slides; 25 males and 8 females kept whole in a vial with 70% ethanol (MZUSP 29083).

##### Derivation of name.

The species is named after the Merendón mountains in Honduras, where the specimens described in the present work were collected.

##### Diagnosis.

Small sized *Elpidium* (c. 700 μm). In dorsal and ventral views carapace relatively elongated for the genus (length/width c. 1,4). In right lateral view carapace elongated (length/height c. 2), with left valve overlapping right valve on all margins, but very strongly at the posterior end of the carapace, especially in females, where this overlap produces a conspicuous outgrowth of the outer lamella, apparently without substantial change to the inner marginal structures. Ventral margin flat, with a subtle ventro-lateral ridge on each valve, at the edge of the flat area. Distal lobe on hemipenis triangular, with a pointed tip and with a finger-like projection at the base of the internal margin next to the dorsal seta; copulatory process a stiff hook-like structure, thick at the first half of its length and then quickly narrowing to the orifice at the tip.

##### Description of male.

Carapace (Fig. 1G–I). Small sized (length = 657–685 μm), with brown surface and sparse setae; surface smooth except for a subtle ventro-lateral ridge on each valve; elliptically elongated in lateral view (length/height=1.96), with greatest height just behind the mid-length; left valve overlapping right valve on all margins, strongly interlocking in antero-ventral, ventral and postero-ventral margins; ventral area flat; dorsal margin arched; posterior and anterior margins rounded, both produced towards the ventral side; oval shaped in ventral and dorsal views, with maximum width behind mid-length; dorsal margin straight in dorsal view; ventral margin sinuous in ventral view with well-marked ridges.

Left valve (Fig. 1A–C). In internal view, anterior and posterior margins rounded, produced towards the ventral side; ventral margin nearly straight; dorsal margin arched; calcified inner lamella well developed anteriorly, with a short line of concrescence near the valve margin leaving a vestibule; vestibule less developed posteriorly, but present; prominent selvage running on all margins except in the middle of dorsal margin; a short flange is formed at the antero-ventral margin; central muscle scars consisting of 4 spots arranged in a vertical row relatively separated from each other and a round small frontal scar.

Right valve (Fig. 1D–F). In internal view, anterior and posterior margins rounded, produced towards the ventral side; ventral margin nearly straight; dorsal margin arched; calcified inner lamella well developed anteriorly, with a short line of concrescence near the valve margin leaving a vestibule; vestibule less developed posteriorly but present; prominent selvage running all around, forming the hinge structures on the dorsal margin; a continuous flange is present, being wide on the antero-ventral margin, very narrow at the mouth region and narrow on the ventral and postero-ventral margins; central muscle scars consisting of 4 spots relatively separated from each other arranged in a vertical row and a round small frontal scar.

Hinge (Fig. 1A–F). A long (c. 3/4 of the valve length) cardinal ridge is present on the right valve, forming at each end, respectively, a small anterior tooth and a large posterior tooth; the ridge is slightly crenulated, especially at the posterior end; RV with complimentary groove and sockets.

Pigmented naupliar eye present; carapace less pigmented at the eye region.

**Figure 2. F2:**
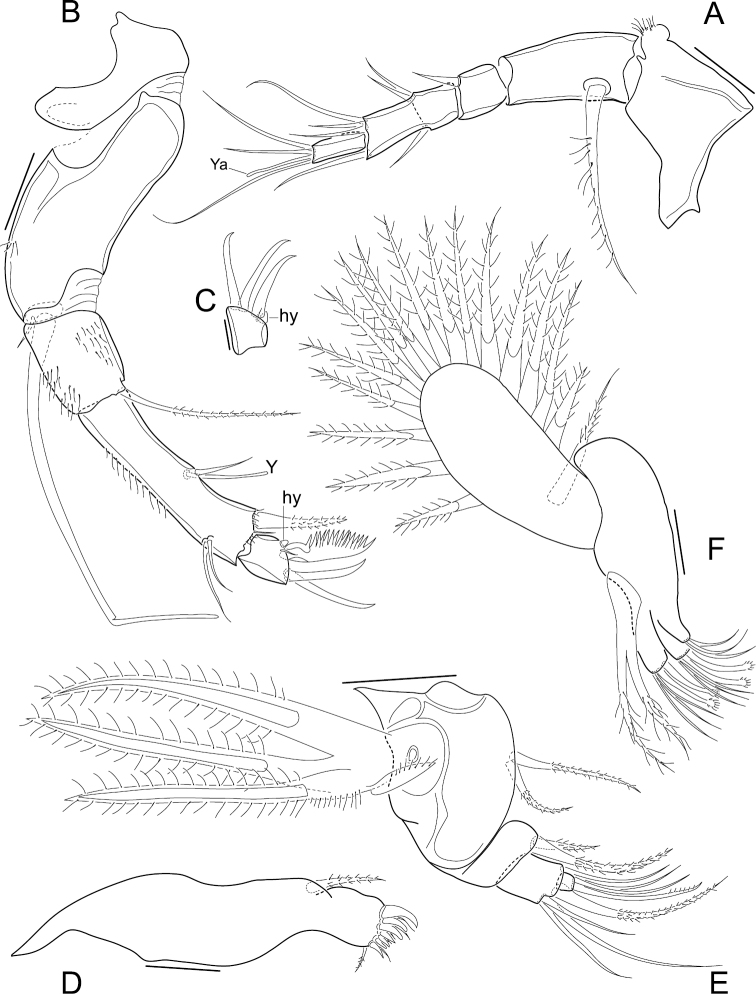
*Elpidium merendonense* sp. n. **A** Antennula **B** Antenna **C** terminal segment of antenna **D** Mandibula **E** Mandibular palp **F** Maxillula **A–B, F** male specimen, holotype, MZUSP 29072 **C** female specimen, allotype, MZUSP 29073 **D–E** male specimen, paratype, MZUSP 29075. Scale bars: **A–B, D–F**: 50 µm; **C**: 20 µm. Ya = aesthetasc on terminal segment of A1. Y = aesthetasc on penultimate segment of antennae. hy = hyaline organ on second antennae.

Antennula (Fig. 2A). 5 functional articles; first article relatively large, bearing on the dorsal margin a sub-apical expansion with a tuft of tiny setules; second article the longest with a ventro-proximal long and thick seta; third article small with a short dorso-apical seta; fourth article partially subdivided in two, medially (where the segment is subdivided) with two dorsal and one ventral setae, and apically with a long ventro-apical, two short and one long dorso-apical setae; fifth (terminal) article with two long setae, one short seta and a short aesthetasc (Ya).

Antenna (Fig. 2B). Protopodite 2-segmented, the first one very short and the second one long, wide and curved; endopodite 3-articulated; first segment relatively short, bearing a long ventro-apical seta; second segment very long and narrow, dorsally with two sub-apical setae, one three thirds as long as the other, ventro-medially with a short seta and an aesthetasc (Y), and apically with two setae, one large and one minute; last segment small, with three claws, the ventral one strongly serrated and the other two slender, a minute seta and a tiny lobe (hyaline formation); exopodite with a very small seta and a spinneret seta.

Mandible (Fig. 2D–E). Coxa with 7 strong teeth and 6 setae on inner edge and a seta on outer edge (near the articulation with the palp); palp 4-segmented (basis + 3-segmented endopodite); basis externally with respiratory plate (exopodite) consisting of 3 rays and one reflexed seta, and internally with two setae, one two thirds as long as the other; first endopodal segment with two apical internal setae, one less than half as long as the other; second endopodal segment with an internal apical seta and 4 external apical setae, one short, two long and one intermediate; terminal endopodal segment with 2 setae and one slender claw, all equally long.

Maxillula (Fig. 2F). Internally with three endites, first one with 2 setae, second and third ones each with 3 setae and two claws, the latter with a conspicuous spoon-shaped apex; palp not segmented, tapering, with 2 apical setae; respiratory plate well developed, carrying a reflexed seta (i.e. reversed towards the front) and 16 long rays.

**Figure 3. F3:**
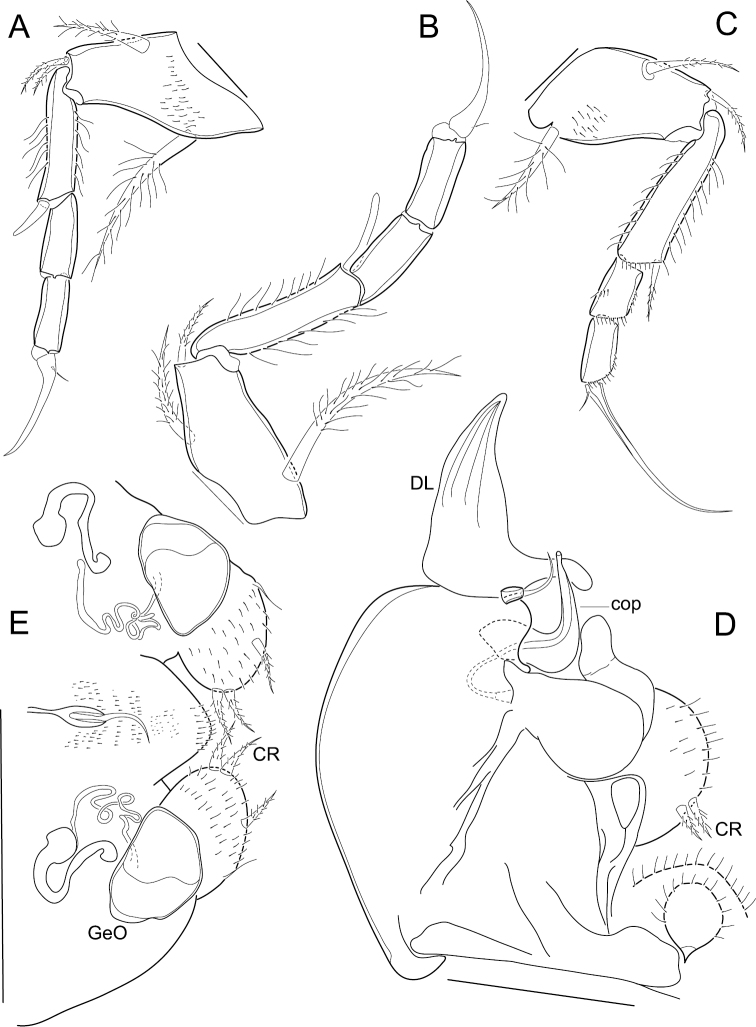
*Elpidium merendonense* sp. n. **A** First thoracic leg **B** second thoracic leg **C** third thoracic leg **D** Hemipenis **E** Abdomen. **A–D** male specimen, holotype, MZUSP 29072 **E** female specimen, allotype, MZUSP 29073. Scale bars: **A–C**: 50 µm; **D**: 100 µm; **E**: 200 µm. DL = distal lobe on hemipenis. cop = copulatory process on hemipenis. CR = caudal ramus. GeO = genital operculum.

First thoracopod (Fig. 3A). 4-segmented; first segment with a long medio-proximal dorsal seta, a medium-sized medio-ventral seta and two stout short ventro-apical setae; second segment quite long, with a strong ventro-apical seta; third segment devoid of setae; terminal segment with an apical claw that bears a minute seta at its base.

Second thoracopod (Fig. 3B). 4-segmented; first segment with a long medio-proximal dorsal seta, a medium-sized medio-ventral seta and a relatively short ventro-apical seta; second segment long, with a strong ventro-apical seta reaching tip of following segment; third segment devoid of setae; terminal segment with an apical claw bearing a minute seta at its base.

Third thoracopod (Fig. 3C). 4-segmented, quite slender; first segment with a medio-proximal dorsal seta, a medio-ventral seta and a ventro-apical seta; second segment quite long, with a slender ventro-apical seta; third segment devoid of setae; terminal segment with a very long and slim apical claw carrying a minute seta at its base.

Hemipenis (Fig. 3D). Consisting of a large rounded muscular body, an articulating distal lobe and a dorsal seta; distal lobe triangular, with a pointed tip and with a finger-like projection at the base of the internal margin, next to the dorsal seta; copulatory process a stiff hook-like structure, thick at the first half of its length and then quickly narrowing to the orifice at the tip; lower ramus (“crochet accessoire”) sinuous and with a rounded tip; caudal ramus a hirsute rounded lobe bearing a pair of setae.

##### Additional description of female.

**Figure 4.**
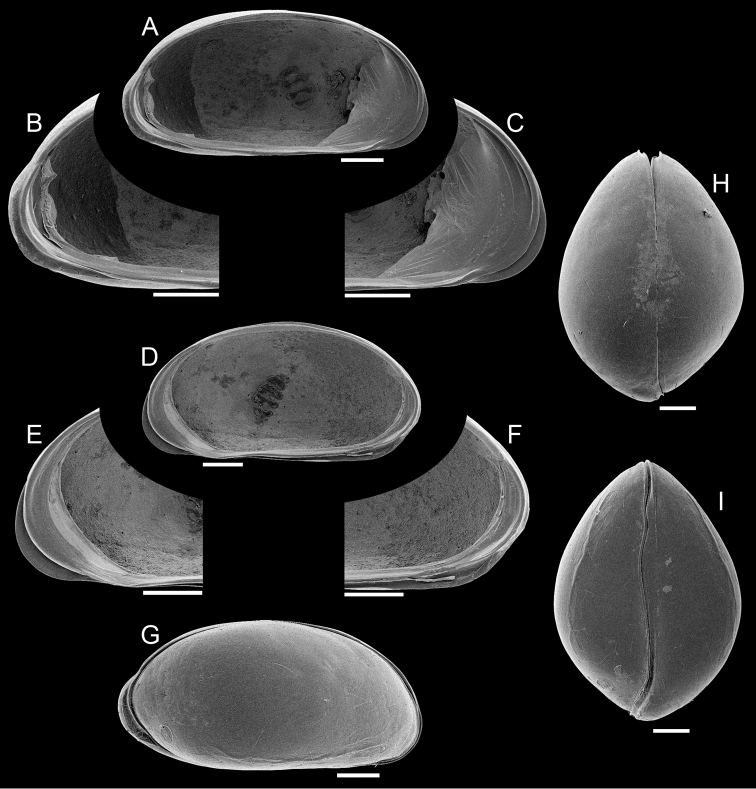
*Elpidium merendonense* sp. n., female. **A** Left valve internal view, general **B** left valve internal view, detail of postero-ventral margin **C** left valve internal view, detail of antero-ventral margin **D** right valve internal view, general **E** right valve internal view, detail of antero-ventral margin **F** right valve internal view, detail of postero-ventral margin **G** right lateral view **H** dorsal view **I** ventral view. **A–F** paratype, MZUSP 29076; **G** paratype, MZUSP 29080; **H** paratype, MZUSP 29081; **I** paratype, MZUSP 29082.Scale bars: 100 µm.

Carapace (Fig. 4G–I). Small sized (length = 697–722 μm), with brown surface and sparse setae; surface smooth except for a subtle ventro-lateral ridge on each valve; elliptically elongated in lateral view (length/height=1.96), with greatest height just behind mid-length; left valve overlapping right valve on all margins, strongly interlocking in antero-ventral, ventral and postero-ventral margins; valve overlap very strong at the posterior end of the carapace, producing a conspicuous outgrowth of the outer lamella, apparently without substantial change to the inner marginal structures; ventral area flat; dorsal margin arched; posterior and anterior margins rounded, both margins produced towards the ventral side; valves oval shaped in ventral and dorsal views, with maximum width displaced towards the posterior end in comparison to the male, producing a brooding cavity; up to 10 eggs were observed in a brood (mean egg size = 4.87 ± 4.0 μm, N = 8); dorsal margin straight in dorsal view; ventral margin sinuous in ventral view with well-marked ridges.

Left valve (Fig. 4A–C). In internal view anterior margin rounded, produced towards the ventral side; posterior margin narrowly rounded, produced towards the ventral side, forming a bulge at the postero-ventral area; ventral margin nearly straight; dorsal margin arched; calcified inner lamella well developed anteriorly, with a short line of concrescence near the valve margin leaving a vestibule; vestibule less developed posteriorly, but present; prominent selvage running on all margins except in the middle of dorsal margin; a short flange is formed at the antero-ventral margin; at the postero-ventral region, with outer lamella expanded towards the posterior end; central muscle scars consisting of 4 spots arranged in a vertical row relatively separated from each other and a round small frontal scar.

Antenna (Fig. 2C). As in the male, except for the small terminal segment with three equally slender claws, a minute seta and a tiny lobe (hyaline formation).

Abdomen (Fig. 3E). Genital operculum rounded, internally connected by tubes to a trabecula; caudal rami two hirsute rounded lobes, each with two apical setae in juxtaposition, a medio-external seta and an inconspicuous external seta nearer the base of the caudal ramus; end of body rounded, with a dorsal seta inserted in a strongly chitinized structure.

##### Measurements.

Male. Holotype: length = 674 µm, height = 347 µm; Paratype MZUSP 29077: length = 685 µm, height = 349 µm; Paratype MZUSP 29078: length = 657 µm, width = 463 µm; Paratype MZUSP 29079: length = 677 µm, width = 461 µm. Female. Paratype MZUSP 29076, LV: length = 721 µm, height = 344 µm; Paratype MZUSP 29080: length = 715 µm, height = 355 µm; Paratype MZUSP 29081: length = 697 µm, width = 513 µm; Paratype MZUSP 29082: length = 722 µm, width = 518 µm.

##### Comparisons.

The carapace of *Elpidium merendonense* sp. n. resembles that of *Elpidium inaequivalve* in being relatively elongated in dorsal view and having a wide valve overlap at the posterior end of the carapace. However, the valve asymmetry is more pronounced in *Elpidium inaequivalve*, while females of *Elpidium merendonense* sp. n. show a unique outgrowth at the posterior end of the left valve where it overlaps the right valve. *Elpidium merendonense* sp. n. and *Elpidium laesslei* both present a distal lobe on the hemipenis with similar shape, but the copulatory process and the lower ramus are smaller in the latter species. These two species can furthermore be distinguished by the shape of the carapace in dorsal view, which is broad and rounded in *Elpidium laesslei* and oval in *Elpidium merendonense* sp. n.

##### Ecology and accompanying fauna.

*Elpidium merendonense* was found only in water accumulated in leaf axils of bromeliads from 1,400 to 2,242m elevation in CNP. Only *Tillandsia* sp. were examined. In this altitudinal range, bromeliads occur all through the area in varying densities depending on microclimatic conditions. Specimens of this species were common in collections throughout the park, sometimes co-occupying bromeliads with a species of candonid ostracod and/or a species of Anomopoda (*Ceriodaphnia laticaudata* Müller, 1867). Ostracoda were more common in larger bromeliads.

## Discussion

Honduras has a complex topography with numerous cloud forested mountain tops characterised by a high endemicity, documented for several taxa such as plants (Bubb et al. 2004), reptiles and amphibians (Wilson and McCranie 2003), and invertebrates (Anderson and Ashe 2000). *Elpidium* species are bromeliad specialists and, considering the high diversity and endemism of *Elpidium* in the molecular study of Jamaican species by Little and Hebert (1996), it is not surprising to find a new species in Honduras. More surprising is that the species described here is the first non-marine ostracod described from inland Honduras (Martens and Behen 1994). Currently, 275 valid species of nonmarine ostracods are recorded from the Neotropical region (Martens et al. 2008), but this figure is highly incomplete. Few localities in this region have been investigated in detail, and large unexplored gaps remain, as is the case with large parts of Honduras. We therefore expect that new sampling campaigns at CNP and elsewhere in Honduras would likely yield more unknown ostracod species, not only from phytotelmata but also from other freshwater habitats.

With the present contribution, seven valid species are currently known in the genus *Elpidium*. Two of these, namely *Elpidium maricaoensis* and *Elpidium laesslei*, are known only from incomplete original descriptions and without records of males, whose reproductive structures bear the most important diagnostic features for identification. Nonetheless, it is still possible to confidently identify all seven species, and an identification key is presented here.

### Identification key to species of the genus *Elpidium*

**Table:** 

1	Carapace surface smooth	2
–	Carapace surface pitted	*Elpidium laesslei*
2	Left valve overlapping right valve	3
–	Right valve overlapping left valve	*Elpidium purperae*
3	Female carapace small to medium size, not longer than 0.8 mm	4
–	Female carapace large, reaching more than 0.9 mm in length	*Elpidium bromeliarum*
4	in dorsal and ventral views, left valve with posterior end markedly longer than right valve, especially in females	5
–	Valves symmetric	6
5	Female carapace in dorsal view with length per width ratio smaller than 1.3	*Elpidium inaequivalve*
–	Female carapace in dorsal view with length per width ratio bigger than 1.35	*Elpidium merendonense* sp. n.
6	female carapace with greatest width at mid-length	*Elpidium pintoi*
–	female carapace with greatest width displaced towards the posterior end	*Elpidium maricaoensis*

All seven known *Elpidium* species were recovered from water impounded inside bromeliads, and distribution of the genus seems to follow almost the whole geographic range of their host plants. *Elpidium* species have been recorded from a variety of altitudes from southernmost Brazil to Cuba: from near sea level (*Elpidium bromeliarum*; see Pinto and Purper 1970), to 1,000 to 1,200 meters above sea level (*Elpidium maricaoensis* and *Elpidium laesslei*, respectively; see Tressler 1956) and up to 2,200 meters above sea level (present study).

The presence of passively dispersing Ostracoda in tank bromeliads requires specialised dispersal vectors. Already in his earliest description of the genus, Müller (1881) discussed possible dispersal mechanisms of bromeliad inhabiting ostracods. Müller (1881) argued that while it may be easy to understand the presence of aquatic insect larvae inside bromeliads, considering that the adults fly away and lay eggs in other plants, the same does not hold for the strictly aquatic ostracods. Müller (1881) hinted on probable phoretic behaviour (i.e. mechanical transportation by other animals) of this species: “they (*Elpidium bromeliarum*) cannot make the necessary travels (from one plant to another) unless they attach to the body of a bromeliad visitor”. More recent field observations (Lopez et al. 1999) and experimental results (Lopez et al. 2005) have confirmed that *Elpidum* can efficiently use frogs and snakes as phoretic dispersal agents to colonise new plants. This ability of *Elpidium* species to hop from plant to plant on phoretic vectors together with the resistance to environmentally harsh conditions such as dehydration (Lopez et al. 2009) are probably the two main traits causing the generally observed dominance of *Elpidium* ostracods inside bromeliads.

## Supplementary Material

XML Treatment for Elpidium

XML Treatment for Elpidium merendonense
